# Effect of provider-initiated testing and counselling and integration of ART services on access to HIV diagnosis and treatment for children in Lilongwe, Malawi: a pre- post comparison

**DOI:** 10.1186/1471-2431-9-80

**Published:** 2009-12-18

**Authors:** Ralf Weigel, Portia Kamthunzi, Charles Mwansambo, Sam Phiri, Peter N Kazembe

**Affiliations:** 1 Lighthouse Trust at Kamuzu Central Hospital, PO Box 106, Lilongwe, Malawi; 2 University of North Carolina Project Lilongwe, Private Bag A104, Lilongwe, Malawi; 3 Kamuzu Central Hospital, Paediatric Department, PO Box 149, Lilongwe, Malawi; 4 Baylor College of Medicine Children's Clinical Centre of Excellence, Private Bag B-397, Lilongwe, Malawi

## Abstract

**Background:**

The HIV prevalence in Malawi is 12% and Kamuzu Central Hospital (KCH), in the capital Lilongwe, is the main provider of adult and paediatric HIV services in the central region. The Lighthouse at KCH offers opt-in HIV testing and counselling (HTC) for adults and children. In June 2004, Lighthouse was the first clinic to provide free antiretroviral treatment (ART) in the public sector, but few children accessed the services. In response, provider-initiated HIV testing and counselling (PITC) and an ART clinic were introduced at the paediatric department at KCH in Quarter 4 (Q4) 2004.

**Methods:**

We analysed prospectively collected, aggregated data of quarterly reports from Q1 2003 to Q4 2006 from HTC centre registers, ART registers and clinic registrations at the ART clinics of both Lighthouse and the paediatric department. By comparing data of both facilities before (Q1 2003 to Q3 2004), and after the introduction of the services at the paediatric department (Q4 2004 to Q4 2006), we assessed the effect of this intervention on the uptake of HIV services for children at KCH.

**Results:**

Overall, 3971 children were tested for HIV, 2428 HIV-infected children were registered for care and 1218 started ART. Between the two periods, the median (IQR) number of children being tested, registered and starting ART per quarter rose from 101 (53-109) to 358 (318-440), 56 (50-82) to 226 (192-234) and 18 (8-23) to 139 (115-150), respectively. The median proportion of tested clients per quarter that were children rose from 3.8% (2.7-4.3) to 9.6% (8.8 to 10.0) (p = 0.0009) and the proportion of ART starters that were children rose from 6.9% (4.9-9.3) to 21.1% (19.2-24.2) (p = 0.0036). The proportion of registered children and adults starting ART each quarter increased similarly, from 26% to 53%, and 20% to 52%, respectively.

**Conclusions:**

Implementation of PITC and integration of ART services within the paediatric ward are likely to be the main reasons for improved access to HTC and ART for children at KCH, and can be recommended to other hospitals with paediatric inpatients in resource limited settings with high HIV prevalence.

## Background

Provider-initiated testing and counselling (PITC) has been identified as a priority intervention to increase access to treatment, care and support for HIV-infected children, since late diagnosis and delayed initiation of antiretroviral treatment (ART) remain prime reasons for their high mortality in resource limited settings[1,2]. Many children living with HIV are still not receiving treatment and many opportunities to diagnose children with HIV are being missed[3]. A number of reviews highlight the need to examine different approaches to increase access for HIV-infected children to care [4-6]. General barriers to scale-up of HTC and ART in children include competing demands of adult care, incomplete utilization of the various entry points into paediatric HIV care, such as HIV testing and counselling (HTC) in paediatric wards and among children of adult ART patients, and poor linkage to prevention of mother-to-child (PMTCT) programmes[2,7,8]. Failure to provide HTC to older children has been identified as a missed opportunity to provide primary prophylaxis and ART[9].

The adult HIV prevalence in Malawi in 2003 was 12.9% and fewer than 3,000 adults and 200 children were receiving ART at this time[10]. An estimated 222,000 adults and 17,600 children under 15 years were in need of ART and initially, children were underrepresented in the national ART programme [10-13].

Here, we examine the uptake of HTC and ART in children before and after the introduction of PITC and ART services at the paediatric department of Kamuzu Central Hospital (KCH), the main hospital in Malawi's capital Lilongwe, with the aim of stimulating the discussion on how to increase access to ART for children in resource limited settings.

## Methods

### Setting

At KCH, HIV services for children are provided at 2 places: at Lighthouse, where HIV-infected children and adults are registered together, and at the paediatric department. Lighthouse is a registered trust at the premises of KCH, and offers a continuum of HIV care and support services through opt-in HTC (voluntary counselling and testing), outpatient clinics and community home-based care[14]. Provision of ART for adults and children started in 2002 and was initially on a cost recovery basis, until ART became free in June 2004[15]. After HTC, HIV-positive children and adults were referred for ongoing care at the Lighthouse clinic, or a general weekly clinic in the paediatric department[16]. Over 13,500 children are admitted to the general wards and the nutritional rehabilitation unit of the paediatric department each year.

PITC for children admitted to the paediatric department, including HTC for their adult caregivers began in October 2004 and was operating daily from one room, with a single counsellor. HIV-infected adult caregivers were referred to Lighthouse and HIV-infected children to the newly established paediatric ART clinic within the paediatric department. This clinic opened in November 2004, operated twice a week and was run buy 2 paediatricians and a nurse[17]. Until the official opening of the paediatric ART clinic in February 2005, ART initiations of all children were recorded at the Lighthouse clinic.

### HIV testing and counselling

Counsellors provide HTC according to the national guidelines and use standardized tools for monitoring and evaluation. The 2003 National HTC guidelines did not specify testing procedures for HIV-exposed children less than 18 months of age, and HIV-DNA PCR tests were not available. In the absence of recommendations, biological mothers were tested to confirm the HIV-exposure status of the child for diagnostic reasons and the children were treated for opportunistic infections. They could be started on ART based on a presumptive diagnosis of severe HIV infection in children under 18 months of age, according to WHO definition and the national ART guidelines[18,19].

All counsellors underwent the national 5-week HTC training. Two counsellors attended an international child-counselling course and trained thereafter the other counsellors. At the Lighthouse, counsellors provide client-initiated HTC for adult and paediatric clients with referrals or drop-in clients. At the paediatric department, counsellors conduct daily sensitisation sessions on HIV-related topics for caregivers of admitted children at the 3 wards and under-5 clinic, highlighting the need for HTC. During ward-rounds, if clinicians and nurses feel HTC for the child is needed, the caregiver is referred to the HTC room. Here, the caregivers (and the older children) receive pre-test counselling, using a checklist, which sensitively guides the counsellor through the process. The caregiver then has the choice to opt-in for HIV testing for the child in question. Over 95% of caregivers give consent for testing at this point. After consent is given, the child undergoes parallel testing with two rapid tests for HIV-1/2 (Uni-Gold™ and Determine™) with a tiebreaker for discordant results (Bioline 3.0™) from whole blood of a finger stick. The caregiver receives post-test counselling, and HIV-infected children are referred with a letter to the paediatric ART clinic for evaluation of ART eligibility. The children themselves are involved in the different steps of this process to a varying degree, depending on their age and understanding.

In order to ensure service quality, there was scheduled fortnightly supervision with in-session observation by the HTC coordinator, twice weekly mentoring between counsellors also with in-session observation, and weekly unannounced spot-supervisions by the coordinator to check availability of counsellors. In addition, there were also weekly meetings with the paediatricians, nurses and counsellors at the paediatric ward to review progress of PITC, and linkage to the ART clinic at the department.

Monthly HTC reports include the number of clients counselled and tested, number of positive results, and a breakdown into sex, adults and children (14 years and below.) Generally, counsellors enquire about the HIV status of the caregiver and recommend HTC, if needed. HTC in adult caregivers follows the same structure of pre- and post-test counselling as described.

### Registration at the ART clinic and ART initiation

HIV-positive clients register at the ART clinics according to their referral letter from HTC with name, age, sex, their HIV status and their referral source. After registration, clinicians treat patients for HIV-related diseases, prescribe Cotrimoxazole prophylaxis and evaluate patients for ART eligibility according to national guidelines (WHO clinical stage 3 and 4, and absolute or relative CD4 count below the age-dependant threshold for severe immunosuppression) [19-21]. After attending a group education session to understand the implications of ART, patients start on a generic fixed dose combination (FDC) of d4T/3TC/NVP. Children use the same FDC at both clinics but split according to weight bands. Upon commencing ART, the patient's details are entered into the national ART register, including age, sex, start date and reason for starting[22]. The Lighthouse treats adults and children, the ART clinic at the paediatric department children only.

### Data collection and analysis

To assess the effect of PITC and provision of ART in the paediatric department (the intervention) we compared quarterly uptake of HTC and ART in the period before the intervention has started (Q1 2003 to Q3 2004 data from Lighthouse only) with the uptake thereafter (Q4 2004 to Q4 2006, data from Lighthouse and paediatric department). For both periods we calculated medians and interquartile ranges (IQR) of the outcome variables: (1) absolute numbers of children tested, registered at the ART clinics and started ART per quarter (2) proportions of clients tested and started on ART per quarter at KCH that were children and (3) proportions of registered children and adults that started ART. To examine the effect of other factors than the introduction of PITC on the uptake of HTC and ART in children we calculated for Lighthouse only (were no PITC is provided) the median proportion of clients tested and starting on ART that were children and compared the results for both periods.

Data were collected prospectively from national HTC registers, registers at clinic receptions and national ART registers, aggregated in monthly and quarterly reports and analysed using Stata (version 10.1, Stata Corporation, College Station, Texas, USA). Proportions, medians and interquartile ranges (IQR) were used to describe the data. Medians of the outcome variables before and after the intervention were compared using the two-sample Wilcoxon rank-sum (Mann- Whitney) test. P- Values of 0.05 or less are considered significant.

### Ethical review

Studies of routinely collected data for national reporting do not require approval from the National Health Science Research Committee of Malawi. Approval for dissemination of these data was given.

## Results

Table 1 gives an overview of children and adults tested, registered and starting on ART between January 2003 and December 2006 at KCH. Over this period counsellors tested 3,971 children and 47,497 adults. Forty-one percent (1,613/3,971) of all children were tested through PITC at the paediatric ward.

**Table 1:** Patients receiving HIV-related services at Kamuzu Central Hospital between 2003 and 2006

	HIV testing and counselling			Registrations at ART clinic			ART initiations		
**Quarter**	**paediatric**		**adult**	**paediatric**		**adult**	**paediatric**		**Adult**
	
**year**	LH*	**dept**.^#^	**LH^§^**	LH	**dept**.	**LH**	**LH**	**dept**.	**LH**

Q1 2003	24	-	1387	23	-	838	6	-	116
Q2	53	-	1484	50	-	778	12	-	116
Q3	70	-	1761	52	-	734	8	-	143
Q4	101	-	2029	82	-	836	23	-	164
Q1 2004	109	-	2431	71	-	966	18	-	205
Q2	126	-	2785	56	-	975	18	-	349
Q3	101	-	3630	129	-	1481	36	-	487
Q4	154	97	3175	112	48	1342	47	-	386
Q1 2005	174	112	3505	102	77	1488	43	-	536
Q2	175	143	3163	113	115	1231	40	75	404
Q3	183	175	3724	75	151	977	51	89	439
Q4	198	155	3328	41	151	769	51	100	636
Q1 2006	167	273	3526	42	192	839	69	125	734
Q2	282	217	3494	35	213	779	22	128	431
Q3	247	196	4116	43	179	781	23	95	453
Q4	194	245	3959	42	234	823	19	120	429

**Total**	**2358**	**1613**	**47497**	**1068**	**1360**	**15637**	**486**	**732**	**6028**

*paediatric services at Lighthouse; ^#^services at the paediatric department of KCH; ^§^services for adults at Lighthouse; Provider-initiated HIV testing and counselling in the paediatric department started in October 2004, ART at paediatric department started operating in November 2004. Until official opening in February 2005, children who started ART at the paediatric department were registered at Lighthouse.

During the same period, 2,428 HIV-infected children under 15 years and 15,637 adults registered in all the ART clinics at KCH. Within the 12 months between Q3 2004 and Q2 2005, 5,542 adults were registered at the Lighthouse, on average 462 per month. Fifty percent of registered children (1,218/2,428) started on ART, 30% (732/2,428) at the paediatric department. Thirty-eight percent (6,028/15,637) of registered adults started on ART.

The median numbers of children taking up HIV-related services per quarter differed significantly between the two time periods before and after the intervention and were 101 (IQR 53-109) and 358 (IQR 318-440) for HIV testing (p = 0.0008), 56 (IQR 50-82) and 226 (IQR 192-234) for ART clinic registration (p = 0.0009) and 18 (IQR 8-23) and 139 (IQR 115-150) for ART initiations (p = 0.0008).

**Figure 1:**
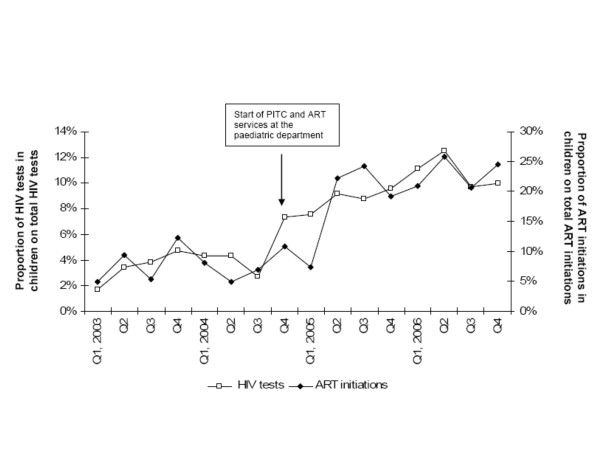
**Trends in proportions of clients tested and patients starting ART that were children**. All children and adults accessing services at Kamuzu Central Hospital (KCH) in each quarter are included. Provider-initiated testing and counselling (PITC) and ART services at the paediatric ward started in Quarter 4 2004.

The proportion of children accessing HTC and ART compared to the total cohort of adults and children increased markedly in Q4 2004 and Q2 2005 respectively (Figure 1). After the intervention, the median proportion of children accessing HTC and starting on ART per quarter, had increased from 3.8% (IQR 2.7-4.3) to 9.6% (IQR 8.8-10.0) (p = 0.0009) and from 6.9% (IQR 4.9-9.3) to 21.1% (IQR 19.2-24.2) (p = 0.0036), respectively.

When the median proportions of clients tested and starting ART that were children were compared for the two time periods for Lighthouse only (where only opt-in HTC is used), the difference was still significant for HIV tests [3.8% (IQR 2.7-4.3) vs. 4.7% (IQR 4.6-5.6), p = 0.005], but not for ART initiations [6.9% (IQR 4.9-9.3) vs. 7.4 (IQR 4.8-9.0), p = 0.87].

**Figure 2:**
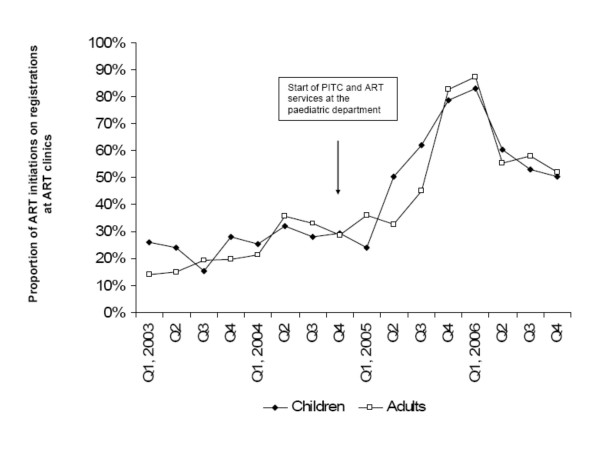
**Trends in proportions of registered children and adults that started ART at the clinics at KCH [at Lighthouse (adults and children) and the paediatric department (children only)]**.

Recruitment in the ART programme increased from Q1 2005 for both, adults and children, since more registered patients started on ART (Figure 2). The median proportion of adults and children starting ART of those registered at the ART clinic differed significantly between the two time periods, and children achieved similar proportions to adults. Before Q4 2004, 26% (IQR 24-28) of registered children and 20% (IQR 15-33) of registered adults started ART. Thereafter, 53% (IQR 50-61) children (p = 0.007) and 52% (IQR 36-58) adults (p = 0.0036) were initiated. The increase in the proportions of ART initiations during the year 2005 was in children mainly due to more ART initiations as registrations remained constant. In adults it was due to constant rates of ART initiation in the context of fewer registrations. During 2006, the proportion of ART initiations on registrations dropped to 50% and 52% in Q4 2006 for adults and children, respectively, because fewer patients started on ART, but these proportions were still higher than in 2003 and 2004.

## Discussion

Our study shows a marked increase in both, the absolute numbers and proportions of children tested for HIV and initiated on ART at KCH in Lilongwe. We attribute this to the utilization of PITC as an additional entry point to the continuum of HIV care. The provision of ART services clearly also provides an incentive for caregivers to consent for their children to be tested.

While an increase in absolute numbers of children accessing HTC and ART can be explained by the availability of free ART since June 2004, the increase in the proportion of children is likely to be caused by the additional entry point at the paediatric department. Several features of PITC contribute to the improved uptake: Firstly, PITC at the paediatric ward carried a high yield. The HIV prevalence of children admitted with fever at the paediatric department of KCH was 30% [23] and of children admitted at nutritional rehabilitation units in Malawi it can be up to 50%[24]. Secondly, the paediatric department is a conducive environment for children, caregivers and providers. Caregivers are usually present, and can be informed and sensitised on HIV and ART individually and in groups. This helps to overcome barriers around HTC for children[25]. Receiving counselling and undergoing testing while already admitted to hospital, does not involve additional cost and time for travelling for patients, and direct referral avoids delay of ART initiation. Thirdly, through PITC, a demand for paediatric HIV services (especially ART) was created and additional resources were mobilized. In 2003 and early 2004, when only opt-in HTC at Lighthouse was used, few children were referred from the paediatric ward and tested compared to adults, and the proportion of registered children who started on ART was much lower, despite the fact that paediatric expertise and ART for children were readily available. As a response to the competing demands of adult care, the paediatric department equipped and staffed an ART clinic, integrated in the routine operations with clear referral structures between PITC and the ART clinic, which finally resulted in a 3-fold increase in the proportion of children starting ART at KCH.

Interestingly, the increase of the proportion of registered adults and children who started ART were tracked together, despite the reasons for the increase differed. PITC and the inpatient ART service allowed the large backlog of eligible children (whose caregivers could not afford ART previously), to be dealt with rapidly. Most of the HIV-infected children identified at the ward were clinically eligible and started on ART without delay, causing the increase in quarterly ART initiations in relation to registrations. Workload at Lighthouse increased dramatically after ART became free in June 2004. The competing demand for adult care at Lighthouse limited the time available to address the specific needs of children and provide high quality care. The new clinic at the paediatric department addressed these needs better and made it an attractive option for both providers and caregivers.

In a pre-and-post comparison, other factors are likely to influence the outcome of interest over time. Even without PITC, access to HTC increased for adults and children at Lighthouse during the study period. Changing attitudes towards HTC among clients and health workers, especially with the availabilityof free ART, are likely to be influencing factors. However, the large increase in the proportion of children accessing ART after Q4 2004 is likely to be related to our intervention. Our study has also other limitations, which are related to the use of aggregated, facility-based data from routine quarterly or monthly reports. In a setting with limited resources, where service provision is a priority, monitoring of patient-level data is difficult. For example, in HTC, clients are registered anonymously. Clients, who come for a second visit to the same institution, are counted twice. However, this may be less relevant in children since they are unlikely to have repeated exposures to HIV requiring a second test. After a positive test, children are registered at the ART clinic and usually start ART. During this process caregivers undergo several sessions of counselling about ART, have accepted the HIV status of their child and will not come for an additional HIV test. We may have also over-reported registrations and ART initiations. Patients who transfer-out at Lighthouse and transfer-in at the ART clinic at the paediatric department are counted twice. We did not examine the degree of referral between HTC and the ART clinics. It is likely that some children, who were tested at KCH, registered and started ART outside KCH and children, who tested outside KCH, came to the ART clinics at KCH. However, the overwhelming majority of children who tested positive at Lighthouse or at the paediatric ward were referred to the respective nearest ART clinic at KCH, since the counsellors work according to a protocol with clear referral guidelines and few other clinics in Lilongwe provided ART to children during most of the period under study. Therefore, it is unlikely that the use of these routinely collected, aggregated data compromises the main conclusions from the study.

Despite the increase in numbers of children tested through PITC, providers initiated HTC in less than 10% of admitted children. No routine PITC system was established, and implementation of a routine PITC opt-out system is challenging[17,26]. Given the low percentage of inpatients undergoing PITC, the potential to increase the utilization of this entry point to care is huge. However, it is also important that other entry points are more fully utilized, including the identification of HIV-exposed children on the postnatal ward and in the under-5 clinic, and building on the success of PITC at ANC[27].

We show, that implementation of PITC at the paediatric ward is feasible, detects many HIV-infected children and, in conjunction with an integrated ART clinic, increases uptake of paediatric ART at the central hospital. However, there is a need to translate the success at central level to the district level, where the proportion of children on ART in most ART clinics is less than 5%[7,28]. When establishing PITC systems for paediatric inpatients, clear referral instructions, and information about ART services are important. ART clinics in district and rural hospitals in Malawi are usually in separate buildings, but run by the staff who provide the general inpatient and outpatient services. Since infrastructure might not allow the establishment of a separate ART service within the ward, general ART clinics should provide family-centred services, which can improve outcomes for children[29].

## Conclusions

Implementation of WHO recommendations on PITC at the paediatric ward at KCH is likely to be the main reason for increased access to HTC for children and, through linkage to an ART clinic in the paediatric department, allowed us to greatly increase the number and proportion of children initiating ART. This model can be recommended to other facilities with paediatric inpatients in resource limited settings with high HIV prevalence.

## Competing interests

The authors declare that they have no competing interests.

## Authors' contributions

RW contributed to data collection, analyzed the data and prepared the manuscript. PK, CM, SP and PNK contributed to data collection, data analysis and reviewed the manuscript. All authors read and approved the final manuscript.

## Pre-publication history

The pre-publication history for this paper can be accessed here:

http://www.biomedcentral.com/1471-2431/9/80/prepub
